# Usability, acceptability, and tolerability of a virtual reality patient tour compared to an educational video in cardiac surgery patients – A feasibility study

**DOI:** 10.1016/j.pecinn.2026.100472

**Published:** 2026-03-18

**Authors:** Cindy M. Cardol, Linda M. de Heer, Marije Marsman, Michelle M. van Rijn, Saskia W.M. Weldam

**Affiliations:** a Nursing Sciences, Program in Clinical Health Sciences, University Medical Centre Utrecht, Utrecht University, the Netherlands; b Department of Cardiothoracic surgery, University Medical Centre Utrecht, the Netherlands; c Department of Anesthesiology, University Medical Centre Utrecht, the Netherlands; d Department of General Practice and Nursing Science, Julius Center for Health Sciences and Primary Care, University Medical Center Utrecht, Utrecht, the Netherlands

**Keywords:** Virtual reality, Cardiac surgical procedures, Patient education, Preoperative care, Feasibility studies

## Abstract

**Objective:**

Two patient educational methods for preoperative cardiac surgery are educational video and Virtual Patient Tour (VPT). We have developed the VPT, the next step is to assess the feasibility. Our aim is to assess the usability, acceptability, and tolerability of the VPT, in comparison with the current standard educational method, the educational video, from a cardiac surgery patients' perspective.

**Methods:**

A single-centre, cross-sectional feasibility was conducted from February to June 2023. The System Usability Scale (SUS), the Unified Theory of Acceptance and Use of Technology (UTAUT), and the Virtual Reality Sickness Questionnaire (VRSQ) were used to assess the usability, acceptability, and tolerability. Data analysis involved descriptive statistics.

**Results:**

Eighteen participants completed questionnaires. The educational video (78.9 ± 14.8) and the VPT (81.8 ± 15.3) both had high SUS scores, indicating high levels of usability. The UTAUT scores (educational video 59.9 ± 5.9; VPT 61.3 ± 5.7) indicated the acceptance of the educational methods. The low VRSQ score (12.5 ± 9.1) indicated that the VPT was tolerable.

**Conclusion:**

The educational video and the VPT were evaluated as usable, acceptable, and tolerable.

**Innovation:**

VPT is a Virtual Reality experience that enhances patients' comprehension of hospital departments by providing a virtual reality experience.

## Highlights

• Virtual Patient Tour (VPT) enhances patients' insight of the hospital divisions.
• VPT and educational video are useful, acceptable, and tolerable education methods.
• VPT can be considered to integrate to the standard care.
• Further research needed to determine the optimal timing of delivering of the VPT.

## 1 Introduction

Cardiac surgery patients often experience preoperative anxiety [1], which may arise from unfamiliar situations, the diagnosis itself, the upcoming anaesthesia, surgery, and the possibility of developing postoperative complications [2], [3]. Preoperative anxiety can negatively impact postoperative recovery [4], including increased postoperative pain and need for more analgesics [5], [6]. These negative postoperative outcomes may be prevented by addressing preoperative anxiety [7]. Preoperative patient education can decrease the patient's preoperative anxiety [8], [9], [10]. Patient education can convey complex information to the patient, which could prepare them for surgery and the recovery process [11], and a better understanding about their hospital stay [3].

Preoperative education can be delivered to patients through various formats, and presenting complex information in different ways can support better understanding and retention [12], [13].Most hospitals use educational videos, information folders and/or a consultation with the nurse and/or doctor to prepare the patient about their upcoming surgical procedure and hospital stay [14], [15]. A more interactive method involves education using virtual reality (VR) [16], [17]. Where videos are two-dimensional to convey information [15], with VR the user can experience a three-dimensional virtual environment using interactive technology, which includes the psychological sense of interacting in the computer world [16], [17]. A VR intervention can be utilised in various ways, including helping patients to comprehend their health state [18], and educating patients [19]. Moreover, patients can become accustomed with an unfamiliar environment through VR, which can help them to understand the surgical process [18], [19]. Therefore, from patients' perspective, using VR is also a promising educational method to increase comprehension and reduce preoperative anxiety [19], [20].

To educate patients undergoing cardiac surgery, a VR-based intervention called the Virtual Patient Tour (VPT) was developed in University Medical Center Utrecht. The patients' needs and perspectives were considered during the development of the VPT [20]. This VPT shows different types of hospital facilities from a patient perspective. In the development of the VR intervention, several models could be used. The Medical Research Council (MRC) framework serves as a guide for the process of developing and evaluating a complex intervention. The MRC framework is structured into multiple phases: development, feasibility and piloting, evaluation, and implementation [21]. Another model, specific in the development of VR interventions is the Virtual Reality Clinical Outcomes Research Expert (VR-core) model [22]. In both models, the feasibility phase is an important step in the development process. The VPT was developed in the development phase, in the subsequent feasibility phase the VPT was tested as usable, acceptable and tolerable [23]. In light of the ongoing innovation in educational techniques, it is important to compare the current educational method with the newly developed VPT to better understand the relative efficacy of the methods. Therefore, in the current study a feasibility study is conducted of the two educational methods with a focus on usability, acceptability, and tolerability [22]. Usability includes the patient's experience in terms of effectiveness, efficiency, and satisfaction [24]. Acceptability indicates the patient's willingness to use the intervention [22]. Tolerability focuses on the tolerance of the intervention [22], as VR can cause symptoms of visually induced motion sickness [25], [26].

The objective of this feasibility study is to assess and to compare the usability and acceptability of the VPT with the current standard of practice, the educational video, in preoperative cardiac surgery patients and to assess the tolerability of the VPT. The results will be used for the next phase of the development of the VPT according to the MRC and VR-core model.

## 2 Methods

### 2.1 Design and context

A single-group, observational, descriptive, and cross-sectional study was conducted using questionnaires to compare the educational video with the VPT. The study was conducted at the anaesthesiology outpatient clinic of a Dutch university hospital. Recruitment occurred between February and May 2023, using consecutive sampling to enrol all patients who met the inclusion criteria throughout the study period [27]. Patients were eligible to participate in the study if they were scheduled for elective cardiac surgery, had preoperative visit to the anaesthesiology outpatient clinic, had a postoperative indication for admission to the intensive and medium care unit after surgery, and were 18 years or older. To participate effectively in the study patients were required to speak, read, and understand Dutch, and be able to provide informed consent. Patients were excluded if they had a visual or auditory impairment and were unable to wear glasses, contact lenses, or hearing aids; had limited arm movement; or had a history of either neurological disease (e.g., epilepsy, cerebrovascular accident, or Parkinson's disease) or vertigo.

### 2.2 Sample size justification

There is no clear recommendation for the sample size for feasibility studies. One study recommended that single-groups studies have a median of 36 participants with a range from 10 to 300 [28]. Another study advised a minimum sample size of twelve participants for single-group feasibility studies [29]. Therefore, this single-group feasibility study aimed for a sample size of 12 to 36 participants, aiming for the highest number of participants.

### 2.3 Products

#### 2.3.1 Educational video

The educational video is a two-dimensional video-based intervention, in which a nurse and a cardiothoracic surgeon describe the preparation for cardiac surgery and what the patient can expect on the day of admission, the day of the operation, the intensive care unit, the medium care unit, the nursing ward, and discharge [14]. The educational video is publicly available on the website of the university hospital [14].

#### 2.3.2 Virtual patient tour

The VPT intervention is designed for patients to immerse themselves in the hospital's locations, such as the nursing ward, holding area, operation room, intensive care unit, and medium care unit. To watch the VPT the PICO Neo 3 Eye VR glasses [30] were used. The VPT is captured from the viewpoint of the patient and utilised a 360-degree virtual environment. Healthcare professionals and a voice-over provided the spoken information in the VPT. The participant can choose to read text balloons that presented additional information about medical devices, and they can determine the pace of their tour through the controller.

### 2.4 Data collection

A cardiothoracic surgeon included patients who were visiting the anaesthesiology outpatient clinic. These patients received a letter inviting them to participate in the study. Those who wished to participate provided written informed consent on the day of their preoperative visit to the outpatient clinic. To avoid potential coercion, consent was obtained by a researcher who was not involved in the patients' clinical care. Next, the researcher collected demographic characteristics, and participants watched first the educational video and then VPT in approximately 30 min. The order of presentation was fixed for all participants. At home, participants completed the questionnaires on the online survey platform (CASTORedc) [31]. If the questionnaire was not completed within a week, a reminder was sent.

#### 2.4.1 Usability

Usability is measured through the System Usability Scale (SUS), a validated tool in the Dutch language [24], [32]. The SUS includes ten questions, rated on a five point Likert scale from ‘strongly disagree’ (1) to ‘strongly agree’ (5) [24].

#### 2.4.2 Acceptability

The Dutch Unified Theory of Acceptance and Use of Technology (UTAUT) is used as aa validated tool for assessing patients' acceptance of an intervention [33], [34]. The questionnaire's core determinants are performance expectation, effort expectation, social influence, and behavioural intention [33]. The UTAUT contains 15 questions, ranked on a 5-point Likert scale ranging from ‘strongly disagree’ (1) to ‘strongly agree’ (5) [35].

#### 2.4.3 Tolerability

Tolerability of the VPT was measured by the Virtual Reality Sickness Questionnaire (VRSQ). The VRSQ is a validated tool assessing nine symptoms of discomfort that people may experience when utilising a VR environment [36], [37]. The VRSQ consists of two concepts, the oculomotor concept, and the disorientation concept. The oculomotor concept includes the following items: general discomfort, fatigue, eyestrain, and difficulty focusing. The disorientation concept subsumes the items: headache, fullness of head, blurred vision, dizzy (eyes closed), and vertigo [36]. A tenth symptom, nausea, was added to the questionnaire by the research team, because nausea can arise as a VR symptom [22], [25], [26]. The VRSQ contains ten questions with a 4-point Likert scale, from ‘none’ (0) to ‘severe’ (3) [36]. The VRSQ was translated into Dutch by the research team. Although care was taken to ensure accuracy, no formal cross-cultural validation of the Dutch version has been conducted.

#### 2.4.4 Evaluation of the interventions

The secondary objective was to evaluate the two educational methods. Several self-designed multiple-choice questions were developed to understand more about the participants' preferences regarding the educational methods, and to gain insight into the tolerability of the two methods. The questions were developed based on the researchers' expertise (Additional File 1).

### 2.5 Data analysis

A descriptive analysis was conducted to summarize the demographic characteristics. Missing data occurred if a participant did not complete the questionnaires. The missing values were accepted when handling with missing data. All analyses were performed with Statistical Package for the Social Sciences (SPSS for 24.0 for Windows).

#### 2.5.1 Usability

The SUS scores were calculated following the method described by Brooke (1995) [24]. The SUS questions includes both positive and negative formulated items. For calculating the SUS, questions (1,3,5,7,9) were deducted one point. Questions (2,4,6,8,10) were scored by 5 minus the scale position. The total scores were multiplied by 2,5. SUS's overall score ranges from 0 to 100 [24]. Usability was classified as poor (≤ 51 points), below average (51–68 points), acceptable (68 points), good (68–80,3 points), or excellent (≥ 80,3) [38], [39].

#### 2.5.2 Acceptability

The mean of the four core determinants of the UTAUT was determined (total score range from 4 to 20). For the comparison of the two educational methods, the total score on the questionnaire was calculated. The researcher team indicated acceptance of the intervention, when the mean value was higher than the scale's midpoint value.

#### 2.5.3 Tolerability

The VRSQ consists of two components, oculomotor and disorientation, with each question ranked from 0 to 3 points, following the calculation method of Kim et al. (2018) [36]. The total of the oculomotor questions (items 1 to 4) was divided by 12, then multiplied by 100. The total of the disorientation questions (items 5 to 9) was divided by 15, then multiplied by 100. The oculomotor score was added to the disorientation score to get the total score, which was then divided by 2 [36].VRSQ score was increased with VR-related discomfort. The VRSQ did not include nausea; therefore, this item was analysed separately and was not included in the total VRSQ score. Nausea was rated on a range of 0 to 3 scale, for which the median was determined.

Two quantitative levels were used to evaluate the usability, acceptability, and tolerability. First, the median and frequency were reported for each question. Second, descriptive statistics, including the mean and standard deviation were provided for the sum of the questionnaires [40], [41].

#### 2.5.4 Evaluation of the interventions

For the preferences and the tolerability of the educational methods, descriptive statistics (frequency, and percentages) were used for the multiple-choice questions.

### 2.6 Ethical issues

The study was conducted in according to the principles of the Declaration of Helsinki and Dutch Regulations. The Medical Research Ethics Committee of the University Medical Centre Utrecht concluded that Medical Research Involving Human Subjects did not apply (register number 212212209). All patients who participated in this study provided written informed consent.

## 3 Results

### 3.1 Participant characteristics

**Fig. 1:**
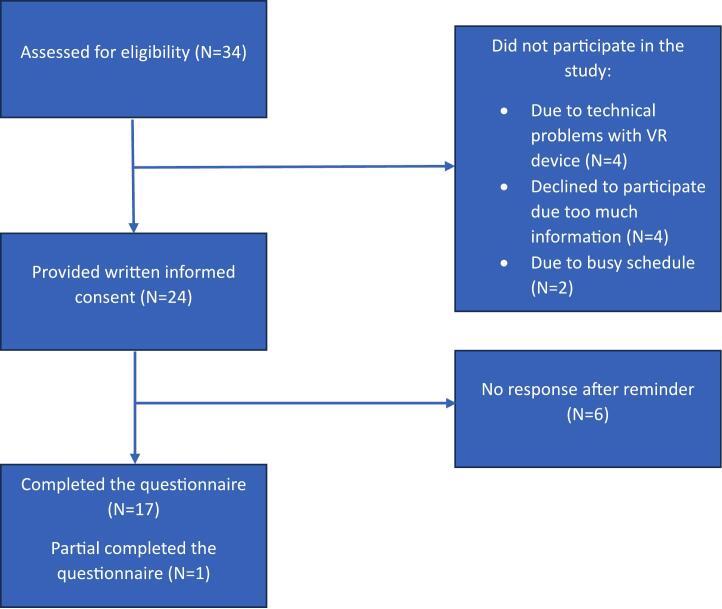
Flowchart participants.

Thirty-four patients were eligible to participate in the study from February 2023 to June 2023. Ten patients did not participate for the following reasons: technical issues with the VR device (*n* = 4), they felt they would receive too much information in one day (n = 4), and a busy schedule (*n* = 2) (Fig. 1). A total of 24 participants provided written consent and were included in the study. The mean age was 66.3 ± 10.0 years, and 54.2% (*n* = 13) were male (Table 1). Most participants had no prior experience with VR technology (*n* = 19). All participants completed the questionnaires before their surgery. The questionnaires were fully completed by 17 participants, and one partially completed. Hence, the final sample size was 18. The response rate was 75.0% (18/24).

**Table 1:** Demographic characteristics of the study population.

Variables		*N* = 24
Age in years (mean ± SD)		66.3 ± 10.0
Sex (n, %)	Male	13 (54.2%)
	Female	11 (45.8%)
Surgical procedure (n, %)	Valvular surgery	10 (41.9%)
	Coronary artery bypass grafting (CABG)	6 (25.0%)
	Combination of valvular and CABG surgery	6 (25.1%)
	Other	2 (8.4%)
Familiarity with Virtual Reality (n, %)	Yes No	5 (20.8%) 19 (79.2%)

SD: standard deviation.

### 3.2 Usability

**Fig. 2:**
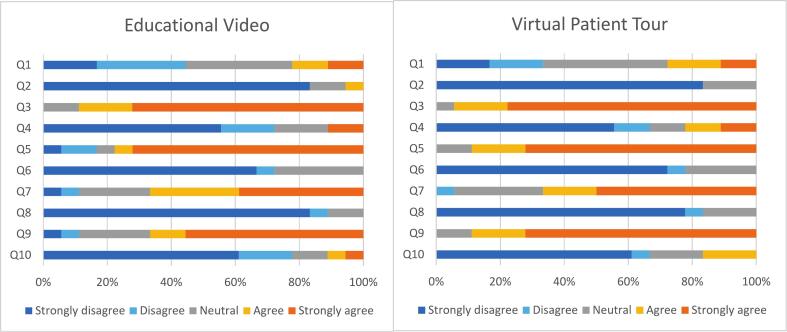
Results of the System Usability Scale (SUS) of the Educational Video and the Virtual Patient Tour (VPT) on question level (in %). Q1: I think that I would like to use ‘intervention’ frequently. Q2: I found the ‘intervention’ unnecessarily complex. Q3: I thought the ‘intervention’ was easy to use. Q4: I think that I would need the support of a technical person to be able to use the ‘intervention’. Q5: I found the various functions in the ‘intervention’ were well integrated. Q6: I thought there was too much inconsistency in this ‘intervention’. Q7: I would imagine that most people would learn to use this ‘intervention’ very quickly. Q8: I found the ‘intervention’ very cumbersome to use. Q9: I felt very confident using the ‘intervention’. Q10: I needed to learn a lot of things before I could get going with this ‘intervention’.

The usability of the educational methods was evaluated using SUS responses. For both educational methods, participants thought the interventions were easy to use (educational video 88.9% (16/18); VPT 94.5% (17/18)). Participants reported feeling confident while using the interventions (educational video 66.7% (12/18); VPT 88.9% (16/18)). Participants reported that they found the various functions in the intervention well integrated (educational video 77.8% (14/18); VPT 88.9% (16/18)) (Fig. 2). Overall, the usability of the educational video was classified as good (78.9 ± 14.8), whereas the VPT was excellent (81.8 ± 15.3).

### 3.3 Acceptability

**Fig. 3:**
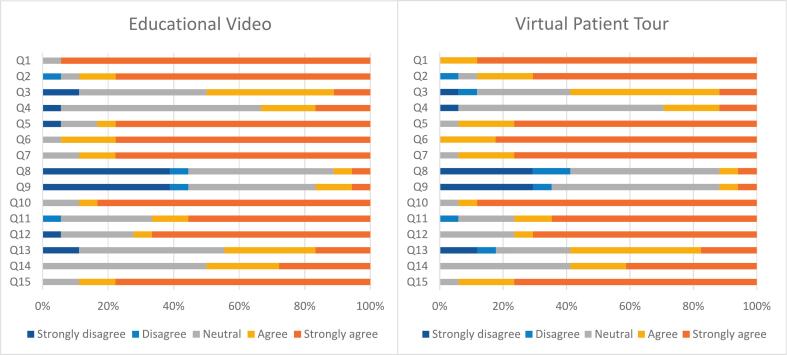
Results of the Unified Theory of Acceptance and Use of Technology (UTAUT) of the Educational Video and the Virtual Patient Tour (VPT) on question level (in %). Q1: I think the ‘intervention’ is a useful tool in the provision of information. Q2: I think the ‘intervention’ helps me to better understand information about the hospital stay and the operation. Q3: The ‘intervention’ helped me to reduce the tension around the operation. Q4: I think that the ‘intervention’ will also give me other positive or negative experiences. Q5: Using the ‘intervention’ costs me little energy and time. Q6: it was easy for me to go through/experience the ‘intervention’. Q7: It was easy for me to learn how to operate the ‘intervention’. Q8: People who influence my behavior thought I should go through the ‘intervention’. Q9: People who are important to me felt it was important for me to go through the ‘intervention’. Q10: I had enough help applying the ‘intervention’. Q11: The UMC Utrecht offers support to view the ‘intervention’. Q12: If the ‘intervention’ is offered before surgery, I would like to experience it. Q13: I would like to experience the ‘intervention’ more than once. Q14: I am interested in experiencing the ‘intervention’. Q15: I would recommend the ‘intervention’ to others.

**Fig. 4:**
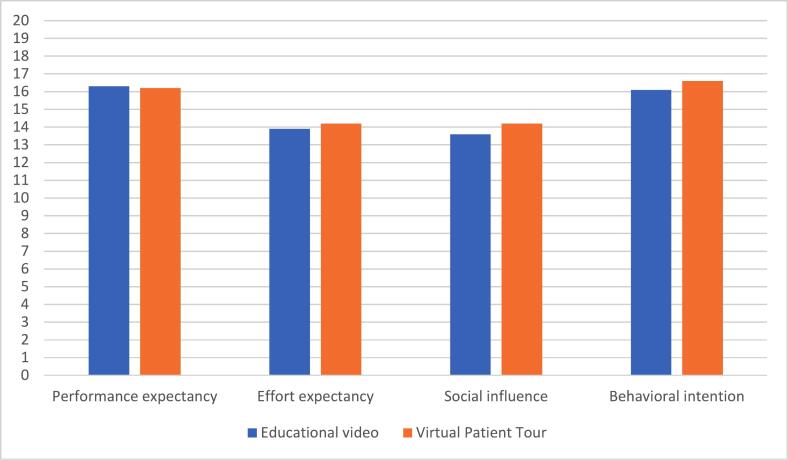
Mean score of Each Determinant of the Unified Theory of Acceptance and Use of Technology (UTAUT) of the Educational Video and the Virtual Patient Tour (VPT).

Responses to the UTAUT were employed to measure acceptability. Participants felt that the intervention helped them to reduce the anxiety around the operation (educational video 50.0% (9/18); VPT 58,9% (10/17)). Participants were interested in experiencing the intervention more than once (educational video 44.5% (8/18); VPT 58.8% (10/17)) (Fig. 3). The mean score per determinant for both educational methods is presented in Fig. 4. The mean values for the educational video were 16.3 ± 2.1 for performance expectancy, 13.9 ± 1.9 for effort expectancy, 13.6 ± 2.8 for social influence, and 16.1 ± 2.9 for behavioural intention. For the VPT, the mean value was 16.2 ± 2.0 for performance expectancy, 14.2 ± 1.2 for effort expectancy, 14.2 ± 2.7 for social influence, and 16.6 ± 2.9 for behavioural intention. The total UTAUT score of the educational video was 59.9 ± 5.9, compared with 61.3 ± 5.7 for VPT, indicating acceptance of both educational methods.

### 3.4 Tolerability

**Fig. 5:**
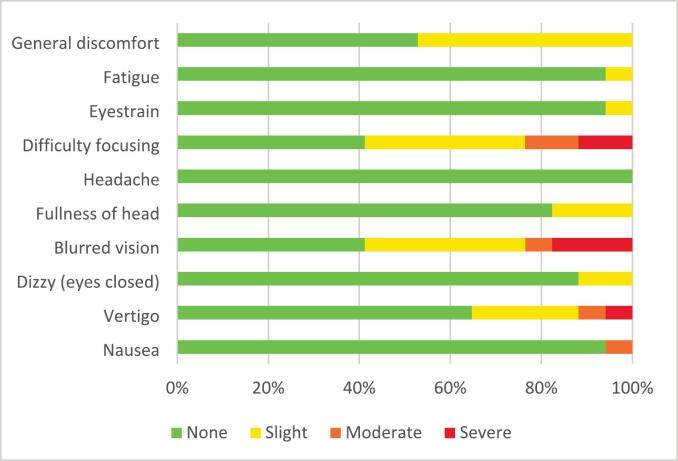
Results of the Virtual Reality Sickness Questionnaire (VRSQ) of the Virtual Patient Tour (VPT) on Symptom level (in %).

Responses to the VRSQ were utilised to assess tolerability. Slight general discomfort was reported by 47.1% (8/17) of the participants, and 58.9% (10/17) of participants reported slight to severe symptoms of difficulty focusing while using VR. Furthermore, 58.8% (10/17) of the participants reported blurred vision as a symptom (Fig. 5). The mean total VRSQ score was 12.5 ± 9.1, indicates that the VPT is tolerable.

### 3.5 Evaluation of the interventions

Half (52.9%, 9/17) of the participants preferred the VPT, whereas a quarter (23.5%, 4/17) reported that the educational video was their preferred method of receiving information in preparation for their hospital stay. Receiving information by both methods was preferred by the remaining quarter (23.5%, 4/17). Nearly all (94.1%, 16/17) of the participants reported that they had watched the entire educational video. Regarding the VPT, all (100%, 17/17) participants answered they had viewed it completely.

## 4 Discussion and conclusion

### 4.1 Discussion

Principal findings.

The purpose of this study was to assess and compare the usability, acceptability, and tolerability of the educational video and the VPT in patients scheduled for cardiac surgery. The VPT was developed using the MRC framework and the VR-core model to provide patients an immersive hospital experience. Both interventions achieved high scores on the SUS and the UTAUT, indicating a high level of usability and acceptability. The relatively low VRSQ score indicated that patients utilising the VPT experienced few negative effects.

Although there is limited literature on the usability and acceptability of an educational video compared to VR for cardiac surgery patients, several findings from the current study are consistent with those of previous research.

The participants rated the educational video as usable and acceptable. Similar findings were observed in a study focussing on a video-based educational intervention for parents and caregivers about their child's asthma [42]. The two studies share some similarities in terms of using educational videos. However, there are notable differences between them, particularly regarding the patient populations. The asthma study targeted parents and caregivers who were educated on managing the child's asthma [42], whereas in the current study, cardiac surgery patients encountered unfamiliar situations related to the surgery. Moreover, the current study employed SUS and UTAUT scores to assess usability and acceptability, whereas the asthma study used a survey on the parental perception on the acceptability, accessibility, utility and usefulness [42]. Despite these differences, in both studies the main conclusion is that an educational video was a usable and acceptable intervention.

The participants in our study rated the VPT as usable and acceptable. These findings are in line with the results of a systematic review on the usability of VR among older adults. In this review adults rated VR as acceptable or marginally acceptable [43]. In several patient groups, such as older adults with cognitive impairment [44], and cardiac surgery patients [23], VR was usable and acceptable. However, there were differences between the current study and previous ones. Firstly, the intervention for cognitively impaired older adults involved immersing the patient in a supermarket [44], whereas the VPT in our study focused on patient education. Secondly, the cardiac surgery patients experienced the VPT on the day before the surgery [23], whereas our participants watched the VPT during their visit to the anaesthesiology outpatient clinic. While our study did not directly measure timing, it was observed that timing was an important aspect. In the study in which the VPT was shown on the day of admission, five participants declined to participate because they were too anxious, too tired, or for other personal reasons [23]. In our study, four participants declined to participate in the study due to the excessive amount of information at that time. Our study showed the educational video and VPT to patients on a fixed schedule, which might not have been optimal for patients to absorb complex information. The optimal moment for patient education in preoperative care is also poorly described in the literature [45]. So, the next step would be to determine the best timing for the patient to receive education. Nonetheless, despite these variations, all the studies provide insight into the usability and acceptability of a VR intervention.

Additionally, the tolerability of the VPT was assessed in this study due to the potential for VR to induce symptoms of visually induced motion sickness [22], [25], [26]. Despite the anticipated risk of nausea as a side effect of VR, most participants reported no signs of nausea. This finding is consistent with the current VRSQ, which no longer includes nausea anymore, because of the low reported incidence [36].

### 4.2 Strengths and limitations

A strength of this study was that consecutive sampling was used, allowing all patients who met the inclusion criteria within the study period to be included [27]. This approach ensured a good representation of the patient population. Another strength was the high response rate [46]. This could be attributed to the participants' increased motivation to engage in the study after watching the educational video and VPT. Additionally, several strategies were utilised to increase the response rate, including giving the participants the option of completing the questionnaires on paper and sending them a reminder [47]. A third strength was that it followed two models that can be used to develop VR interventions. The feasibility study was in line with the MRC framework and VR-core model for the development of the VPT [21], [22]. Following these models will make the development of an intervention more efficient and tailored. The models also aid in determining whether the research should advance to the next phase, revert to the previous phase, or be halted [21].

Some limitations need to be considered. First, the included number of participants was reached, but due to technical issues with the VR device, four patients could not participate in the study. This underlines the need to address these issues before the VPT can be used in practice. Second, despite the study's focus on the usability and acceptability of the educational video and VPT, the researcher directly provided the participants with the educational video, bypassing the steps that would normally be required to access the educational video. This may have influenced the participants' responses on the SUS and the UTAUT, with participants desired responses rather than reporting their actual experiences. However, all participants were familiar with the use of computers, so this may be negligible. Third, the educational methods were presented in a fixed sequence, with the standard method shown first, followed by the VPT. This may have influenced participants' perception of the VPT due to increased familiarity with the content. Fourth, the researcher remained in the same room as the participant during the educational video and VPT to be available for any questions. This presence may have influenced how the educational video and VPT were used. To minimize socially desirable responses, the questionnaires were completed outside the researcher's presence. Fifth, a limitation of this study is that the VRSQ questionnaire, while translated into Dutch, has not undergone formal cross-cultural validation. Future research should aim to validate this instrument in Dutch-speaking populations to strengthen the reliability and generalizability of the findings. Lastly, the VR-core model recommends investigating into the reasons why some patients decide not to participate in the intervention [22]. The timing of the VPT delivery may be an essential factor in this.

### 4.3 Recommendation for future research

According to the MRC framework and the VR-core model, the next phase of the VPT development is evaluation [21], [22]. However, an evaluation study is not recommended, due to the high costs associated with evaluation studies and the minimal additive value the results would contribute to the development of the VPT [48]. In this study, only one predetermined moment was available for participants to watch the VPT. Some patients declined to participate due to feeling overwhelmed with the information received that day. Therefore, future research should focus on cardiac surgery patients' preferences for the moment in time when they watch the VPT.

### 4.4 Innovation

Preoperative education can help to reduce anxiety and contribute to improved postoperative outcomes [11]. Moreover, variations in the delivery of preoperative information have been shown to enhance both patients' understanding and their ability to retain the information [12], [13]. Standard preoperative methods, such as educational videos, information leaflets, and consultations with healthcare professionals, provides essential information about the upcoming surgery. Integrating VR into preoperative education not only reinforces this information through an different method, but also allows patients to become familiar with the hospital environment prior to admission [18], [19].

The developed VPT is an innovation tool that allows the patients to immerse themselves in various hospital locations as part of their preparation for cardiac surgery, providing more informed guidance prior their hospital stay [20], [23]. The VPT aligns with the ongoing developments in educational methods. Whereas the healthcare providers primarily provides medical information, the VPT adds an visual experience that helps the patients comprehend their hospital stay.

Based on this study, no recommendation can be made regarding which of the two educational methods is more usable or acceptable for cardiac surgery patients. Both educational methods may be usable, acceptable and tolerable for cardiac surgery patients, so both may be offered to patients, so individual patient can choose their own preference. Therefore, the VPT could be considered for integration into the standard care, once the technical issues with the VR device are resolved.

### 4.5 Conclusion

This feasibility study was part of the development of the VPT and compared the VPT with the educational video in patients scheduled for cardiac surgery. Participants found both the educational video and the VPT useful and acceptable. Furthermore, the VPT was well tolerated, with minimal adverse effects. Based on the findings, both the educational video and the VPT are usable, acceptable, and tolerable educational methods for cardiac surgery patients. Future research should concentrate on understanding patients' preferences regarding the timing of their VPT experience.

## CRediT authorship contribution statement

**Cindy M. Cardol:** Writing – review & editing, Writing – original draft, Project administration, Methodology, Formal analysis, Data curation, Conceptualization. **Linda M. de Heer:** Writing – review & editing, Supervision, Resources, Conceptualization. **Marije Marsman:** Writing – review & editing, Resources. **Michelle M. van Rijn:** Writing – review & editing, Methodology. **Saskia W.M. Weldam:** Writing – review & editing, Supervision, Resources, Methodology, Conceptualization.

## Funding sources

None.

## Declaration of competing interest

The authors declare that they have no known competing financial interests or personal relationships that could have appeared to influence the work reported in this paper.

## Appendix A Supplementary data

Supplementary material: Evaluation of the educational methods - Questionnaire Image 1
