# Supplementary material for: Usability, acceptability, and tolerability of a virtual reality patient tour compared to an educational video in cardiac surgery patients – A feasibility study
Source: PEC Innov. 2026 Mar 18;8:100472. doi: 10.1016/j.pecinn.2026.100472 (PMC13020066; doi:10.1016/j.pecinn.2026.100472)
Supplement: Supplementary file 1 — Supplementary material: Evaluation of the educational methods - Questionnaire [file mmc1.docx]

Additional File 1

Evaluation of the educational methods – questionnaire

Below are some additional questions about the content of the educational video and the VPT.

1. In which of the following method would you prefer to receive information in preparation for admission for cardiac surgery?

☐ Educational video

☐ VPT

☐ Other, namely:

1. Why do you prefer this method?
2. Were you able to watch the entire educational video?

☐ Yes

☐ No (please continue to question 4)

☐ Yes, with a (short) interruption (please continue to question 4).

1. If not, what was the reason for stopping or pausing the educational video?
2. What did you think of the educational video?
3. Were you able to experience the entire VPT?

☐ Yes

☐ No (please continue to question 7)

☐ Yes, with a (short) interruption (please continue to question 7).

1. If not, what was the reason for stopping or pausing the VPT?
2. What did you think of the VPT?
